# Numerical investigations on the strain-adaptive bone remodelling in the periprosthetic femur: Influence of the boundary conditions

**DOI:** 10.1186/1475-925X-8-7

**Published:** 2009-04-16

**Authors:** Bernd-Arno Behrens, Ingo Nolte, Patrick Wefstaedt, Christina Stukenborg-Colsman, Anas Bouguecha

**Affiliations:** 1 Institute of Metal Forming and Metal-Forming Machines, Leibniz Universität Hannover, Garbsen, Germany; 2 Small Animals Clinic, University of Veterinary Medicine Hannover, Hannover, Germany; 3 Department of Orthopaedics, Hannover Medical School, Hannover, Germany

## Abstract

**Background:**

There are several numerical investigations on bone remodelling after total hip arthroplasty (THA) on the basis of the finite element analysis (FEA). For such computations certain boundary conditions have to be defined. The authors chose a maximum of three static load situations, usually taken from the gait cycle because this is the most frequent dynamic activity of a patient after THA.

**Materials and methods:**

The numerical study presented here investigates whether it is useful to consider only one static load situation of the gait cycle in the FE calculation of the bone remodelling. For this purpose, 5 different loading cases were examined in order to determine their influence on the change in the physiological load distribution within the femur and on the resulting strain-adaptive bone remodelling. First, four different static loading cases at 25%, 45%, 65% and 85% of the gait cycle, respectively, and then the whole gait cycle in a loading regime were examined in order to regard all the different loadings of the cycle in the simulation.

**Results:**

The computed evolution of the apparent bone density (ABD) and the calculated mass losses in the periprosthetic femur show that the simulation results are highly dependent on the chosen boundary conditions.

**Conclusion:**

These numerical investigations prove that a static load situation is insufficient for representing the whole gait cycle. This causes severe deviations in the FE calculation of the bone remodelling. However, accompanying clinical examinations are necessary to calibrate the bone adaptation law and thus to validate the FE calculations.

## Background

For the treatment of advanced degenerative or traumatic damages of hip joints, total hip arthroplasty (THA) is well proven [1]. Nevertheless, due to the different mechanical properties of the prosthesis material and the bone tissue a partial unloading of the periprosthetic bone occurs. This phenomenon is called stress shielding [2]. Hypothesized by Wolff's law [3] the bone adapts to the load decrease in consequence of stress shielding by resorption. Thus, an aseptic loosening of the implant arises [4]. Hence, bone remodelling and especially bone resorption is a crucial cause of aseptic loosening of hip prostheses [5].

By now, there are several works dealing with numerical [6-13] and experimental [4,14,15] investigations on bone remodelling after THA. In these numerical studies by means of the finite element analysis (FEA), certain boundary conditions were defined.

According to Morlock et al. [16], walking is the most frequent dynamic activity of a patient after THA. Thus, researchers usually studied one [2,11,17,18] (or a maximum of three [8,9,19]) static load situation(s) of the gait cycle in order to compute the changes in the physiological strain distribution after THA or bone remodelling. Speirs et al. [20] showed that the constraints play an important role for numerical load computations at the femur.

The study presented here investigates the influence of the load situation, in particular whether it is useful to consider only one static load situation of the gait cycle in the FE calculation of the bone remodelling after THA.

**Figure 1:**
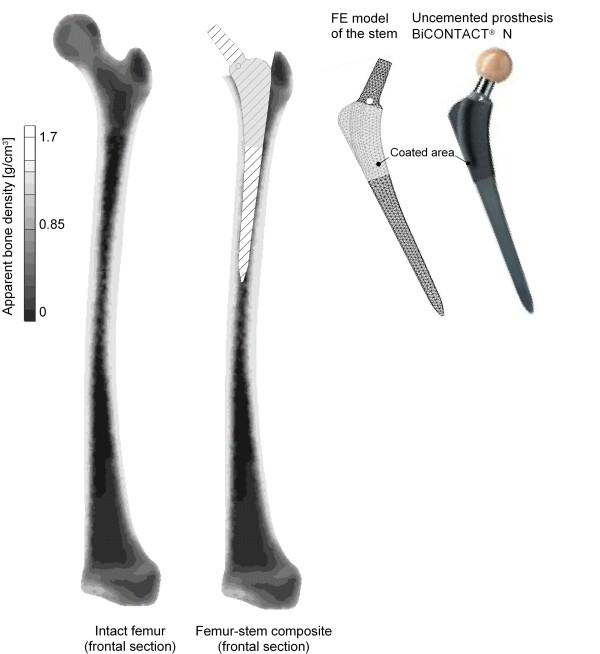
**FE model of the intact femur (left), the composite (middle) and the stem used (right)**. This figure shows on the left side the distribution of the ABD in the frontal section of the intact femur. In the middle the frontal section of the composite femur-stem is illustrated. On the right side an image and the FE model of the conventional uncemented BiCONTACT^® ^N prosthesis are presented.

The prosthesis used for these investigations was BiCONTACT^® ^N (AESCULAP AG, Tuttlingen, Germany), a conventional uncemented (anchored by the "press fit" procedure) stem, see figure 1 (right). This implant is commonly used for the treatment of degenerative wear or high-grade dysplasias of hip joints in the Department of Orthopaedics of the Hannover Medical School (MHH).

## Materials and methods

### Modelling

For the numerical investigations presented here, a STL (Standard Triangulation Language) model of the left femur based on CT data of a male patient with 85 kg weight was generated by means of the 3D medical image processing and editing software Mimics (Materialize, Leuven, Belgium). The CT data were collected in preparation for robot-assisted THA. After informed consent was obtained, the caudal pelvis and the femur were scanned with a slice thickness of 2 mm.

By using the pre-processor software HyperMesh (Altair Engineering GmbH, Böblingen, Germany), a meshed solid model was generated. This model was meshed using ten-noded tetrahedral elements.

The distribution of the apparent bone density (ABD) was first calculated from the measured Hounsfield Unit (HU) values according to Eq. 1 [21] and then translated into the FE model, also using Mimics.

(1)

To couple the Young's modulus of the bone with the ABD, the relationship between these two terms is described as a power function according to Eq. 2 on the basis of the experimental study of Carter et al. [22].

(2)

The distribution of the ABD in the frontal section of the intact femur is shown in figure 1 (left).

The modelling of this composite, as shown in figure 1 (middle), was done with the preprocessor HyperMesh, and it was verified with the manufacturers' OP instructions and the available radiographs from the patient data of the Department of Orthopaedics of the MHH. The computer aided design (CAD) data of the BiCONTACT^® ^N femur component was provided by the producer.

The prosthesis is made of the titanium alloy TiA6V4, and the proximal area of the stem is coated with pure titanium powder applied in a plasma spray process under vacuum conditions (Plasmapore^®^). The Plasmapore^®^/titanium coating has an overall thickness of 0.35 mm and a microporosity of 35%. The pore size varies between 50 and 200 *μ*m.

In the FE modelling a homogenous and isotropic material law (*E *= 110,000 N/mm^2^) is used for the prosthesis, and the coating is regarded by different friction coefficients in the contact region between bone and stem for the proximal and distal areas.

### Principle for the FE calculation of strain-adaptive bone remodelling

**Figure 2:**
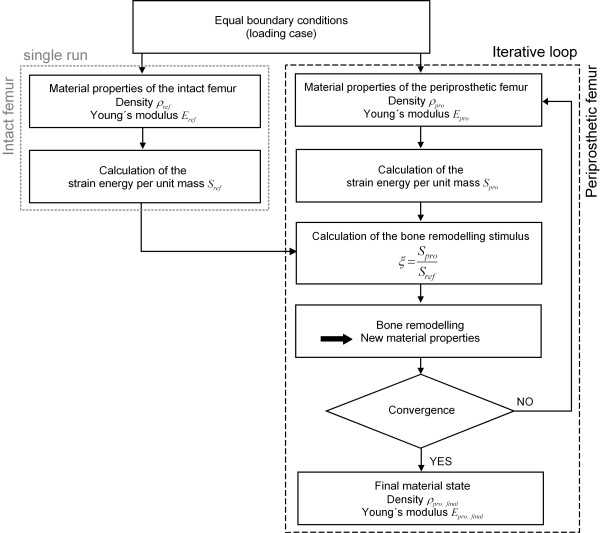
**Principle for the numerical computation of the bone remodelling after THA**. The strain-adaptive bone remodelling in the periprosthetic femur was computed using the FEA. *Figure 2 *shows the principle of this procedure. The physiological load distribution in the intact femur according to a specific loading case was computed in one single cycle. For this, the strain energy per unit of mass *S *was calculated and serves as the reference data to compute the strain-adaptive bone remodelling. After THA, the load distribution in the periprosthetic femur, according to the same loading case as in the intact one, changes. In each computation step the stimulus *ξ *for the bone remodelling is calculated. Using the bone adaptation law, the new material properties of the bone structure in the femur after THA were determined for the next computation step. This is an iterative process, in which the simulation ends when convergence and thus the stationary state are reached. The computed elastic properties (*ρ*_end _and *E*_end_) of the bone in this final state are supposed to correspond to the periprosthetic femur's real long-term situation.

Within this study, the strain-adaptive bone remodelling in the periprosthetic femur was computed by means of the FE solver MSC.Marc (MSC.Software Corporation, Santa Ana, USA) according to the principle below (figure 2).

The physiological load distribution in the intact femur according to a specific loading regime (joint and muscle forces) was computed in one single cycle. For this, the strain energy density *D *was calculated according to Eq. 3.

(3)

Herein,  represents the strain vector and  the transposed stress vector. From these, the strain energy per unit of mass *S *is determined (Eq. 4).

(4)

These results serve as the reference data to compute the strain-adaptive bone remodelling. After THA, the distribution of the physiological load in the periprosthetic femur, according to the same loading regime as in the intact one, changes. The stimulus *ξ *for the bone remodelling is defined by the ratio of the strain energy per unit of mass in the periprosthetic femur *S*_*pro *_to that in the physiologically intact one *S*_*ref *_(Eq. 5).

(5)

As the next step, the new material properties of the bone structure in the femur after THA were determined. This is an iterative process, in which the simulation is ended when convergence is reached. To define a convergence criterion, the average ABD in the periprosthetic femur  is computed at the end of each computation step *n *according to Eq. 6.

(6)

Herein, *N *represents the number of elements in the FE model of the periprosthetic femur. Convergence is reached when the difference between average density in the prosthetically treated femur between two steps *n *- 2 and *n *- 1 (), as well as *n *- 1 and *n *(), fulfils the following condition (Eq. 7):

(7)

By this, a stationary state is reached. The computed elastic properties of the bone in this state (*ρ*_end _and *E*_end_) are supposed to correspond to the periprosthetic femur's real long-term situation.

### Bone adaptation law

In order to determine the ABD evolution in the femur after THA, a modified version of Huiskes' bone adaptation law [9,10] was used.

Huiskes et al. assumed that the bone adaptation rate  and the bone modelling stimulus *ξ *correlated linearly with each other [9,10]. Furthermore, they introduced the dead zone *z*. In this zone, changes in the physiological load situation do not cause remodelling processes [9,10]. The threshold level used in this study was *z *= 75%.

**Figure 3:**
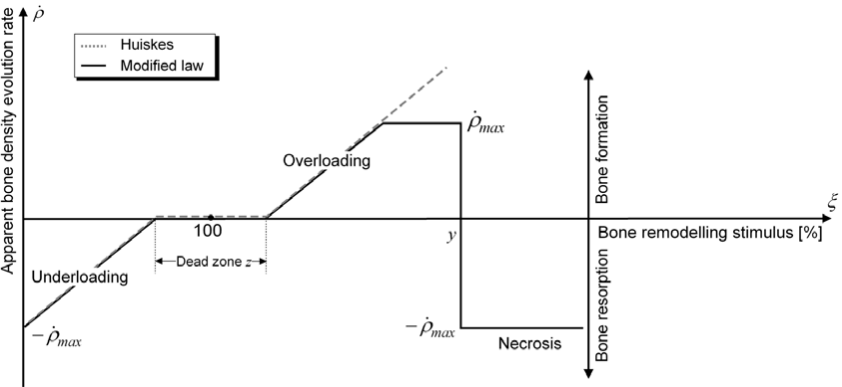
**Modified bone adaptation law**. In this figure the bone adaptation law of Huiskes with the conducted modifications is shown. Here, the correlation between the ABD evolution rate and the bone remodelling stimulus is presented. Huiskes et al. supposed that the bone adaptation rate  and the bone modelling stimulus *ξ *correlated linearly with each other [18]. Furthermore, they used the dead zone *z*, in which changes in the physiological load situation do not cause remodelling processes. In the modified law, we introduced a limitation of the bone formation and assumed that severe overloading causes a necrosis in the bone structure and thus bone resorption.

In the modified law, it is assumed that the bone formation rate must not exceed the maximum value of the resorption rate [5,23] and that, according to biomechanical observations [3], severe overloading (*ξ *> y) causes a necrosis in the bone structure and thus bone resorption [23] (figure 3). Here, the threshold level used was *y *= 400%.

In addition, as indicated by biomechanical examinations, the ABD must not exceed a maximum value of 1.7 g/cm^3^, and, out of numerical considerations, the ABD must not equal 0 because otherwise it would not be possible to calculate the strain energy per unit of mass *S *according to Eq. 4.

### Boundary Conditions

#### Constraints

**Figure 4:**
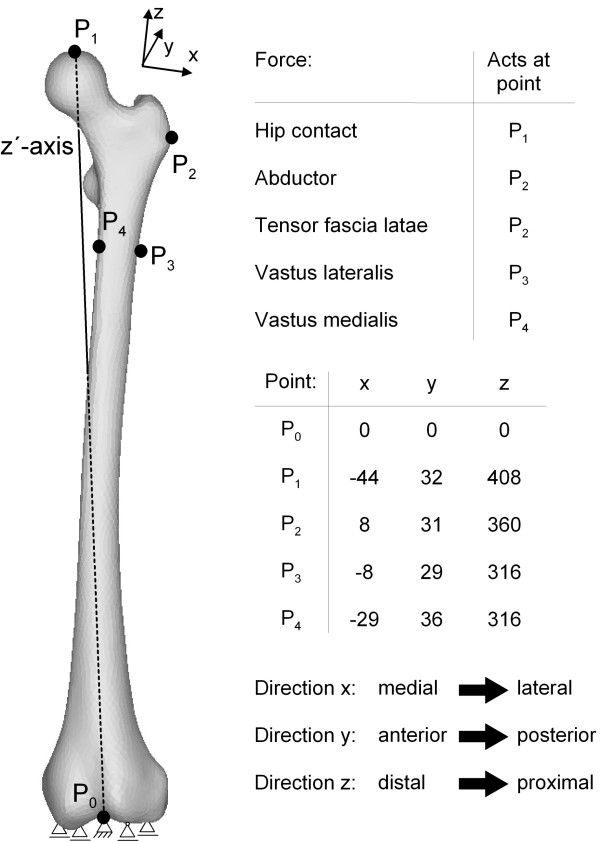
**Boundary conditions (constraints and force acting points) shown for the intact femur (taken from **[20,27]**)**. This figure shows the constraints used for the femur. In the distal condyle, the central node P_0 _was fixed using one thrust bearing, and four other nodes were constrained with floating bearings. Also, the acting node P_1 _of the hip contact force was constrained according to Speirs et al. [20] such that this node can only move along the z'-axis towards the centre of the distal condyle (P_0_). In addition, the used reduced muscle system with the acting points of the forces according to Heller et al. [27] is presented in this figure. It consists of abductors (M. *gluteus minimus*, M. *gluteus maximus *and M. *gluteus medius*), the M. *tensor fascia latae*, the M. *vastus medialis *and the M. *vastus lateralis*.

To approximate the physiological conditions of the femur, the following constraints were chosen according to Speirs et al. [20]. In the distal condyle, the central node P_0 _was fixed using one thrust bearing, and four other nodes were constrained with floating bearings. Furthermore, the node P_1_, where the hip contact force was applied, was constrained such that this node can only move along the z'-axis towards the centre of the distal condyle (P_0_), as shown in figure 4.

#### Loads

Five different loading cases were investigated in this study. First, the four different static loading cases A, B, C and D at 25%, 45%, 65% and 85% of the gait cycle, respectively, and then the whole gait cycle in the loading regime E were examined in order to regard all the different loadings of the cycle in the simulation.

In this regime E, all forces on the femur are discretized by the time with a period of 0.05 s, which corresponds to a frequency of 20 Hz. This yields *l *= 23 different static loadings, each with an individual strain energy density *D*_*l*_, from which the total strain energy density *D*_*total *_is calculated, Eq. 8.

(8)

Accordingly, Eq. 4 is modified into Eq. 9.

(9)

**Figure 5:**
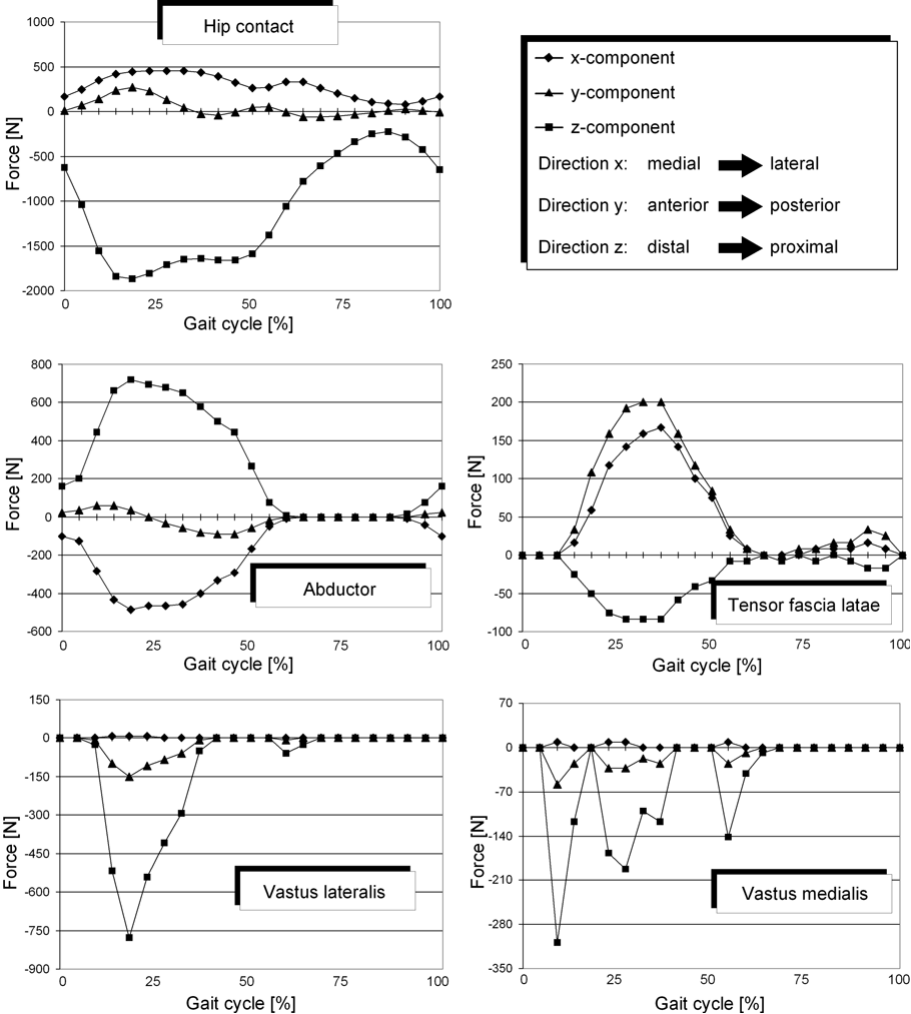
**Evolution of hip contact and muscle forces during the gait cycle (composed from **[28,29]**)**. In this figure the progress of the hip contact and the regarded muscle forces of the whole gait cycle measured by Bergmann et al. [18] and Duda et al. [29] are presented. These data were used for the loading regime E.

As former numerical studies [6,24-26] had shown the influence of the muscle forces on the load distribution and on the computation of the bone remodelling, a reduced muscle system according to Heller et al. [27] was used. It consists of abductors (M. *gluteus minimus*, M. *gluteus maximus *and M. *gluteus medius*), the M. *tensor fascia latae*, the M. *vastus medialis *and the M. *vastus lateralis*. The acting points of the hip contact and the muscle forces are shown in figure 4. The progress of the hip contact and muscle forces during the gait cycle are taken from Bergmann et al. [28] and Duda et al. [29]. For the loading regime E the forces are presented in figure 5 and in Table 1 for the loading cases A, B, C and D.

**Table 1:** Hip contact and muscle forces in the static loading cases A, B, C and D

Loading case	A (25% gait cycle)			B (45% gait cycle)			C (65% gait cycle)			D (85% gait cycle)		
Component	x	y	z	x	y	z	x	y	Z	x	y	z

Hip contact	451.4	225.7	-1806	393	-41.8	-1663	334.4	-58.5	-786	-108	-16.7	-251

Abductor	-468	0	694	-334	-92	501.6	0	0	0	0	0	0

Tensor fascia latae	117	158.8	-75.2	142	158.8	-58.5	0	0	0	8.4	16.7	0

Vastus lateralis	8.4	-108	-543	0	0	0	0	0	-25	0	0	0

Vastus medialis	8.4	-33.4	-167	0	0	0	0	0	-8	0	0	0

In this table the values of the hip contact and muscle forces for the static loading cases A, B, C and D at 25%, 45%, 65% and 85% of the gait cycle respectively are presented. This measured data is according to Bergmann et al. [28] and Duda et al. [29].

## Results

**Figure 6:**
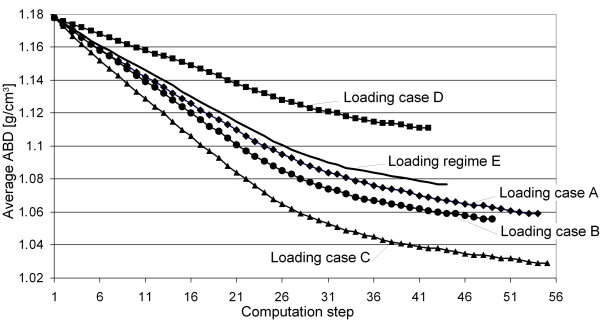
**Progress of the average ABD over the computation steps for the five loading cases**. Here, the evolutions of the average ABDs in the periprosthetic femur for all five examined loading cases (four static loading cases A, B, C and D plus the kinematic loading regime E, in which the whole gait cycle was examined) are presented. This figure shows that – however, the evolutions of the average ABDs are qualitatively similar – the simulations converge at different computation steps and that the final average ABDs after reaching convergence differ.

**Figure 7:**
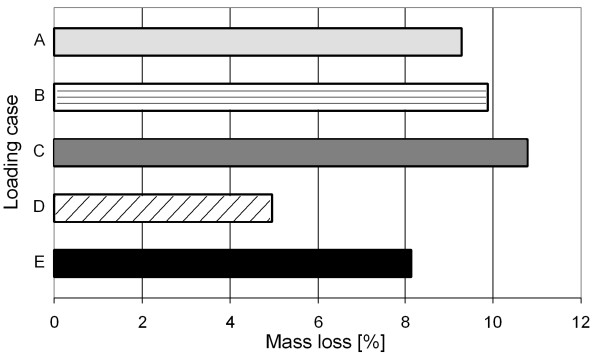
**Computed mass losses for the five loading cases**. This figure shows the calculated mass losses due to the different loading cases. These mass losses take the values 9.27%, 9.87%, 10.79%, 4.97% and 8.15% for the loading cases A, B, C, D and E respectively.

In figure 6, the progress of the average ABD in the periprosthetic femur for all five examined loading cases is presented. It can be seen that all five simulations converge at different computation steps. Qualitatively, the progress of each average ABD in all five simulations is similar, and only the final average ABDs after reaching convergence differ. This indicates varying mass losses due to the different loading cases, as shown in figure 7.

The mass loss in the computation using loading case D is underestimated because it represents the minimum loading on the femur. The deviation compared to the mass loss computed with loading regime E is 40%.

Using loading case A, the computation yields more bone mass loss because this case represents the maximum loading on the femur. The deviation from loading regime E is 14%.

Although the loading on the femur decreases in B and C, there is increased mass loss compared to the computation with A, and it is also overestimated in comparison to loading regime E. This is because the muscle forces in these loading cases are rather small, see Table 1. For these cases, the deviations from the mass loss computed with E are 21% (loading case B) and 35% (loading case C), respectively.

**Figure 8:**
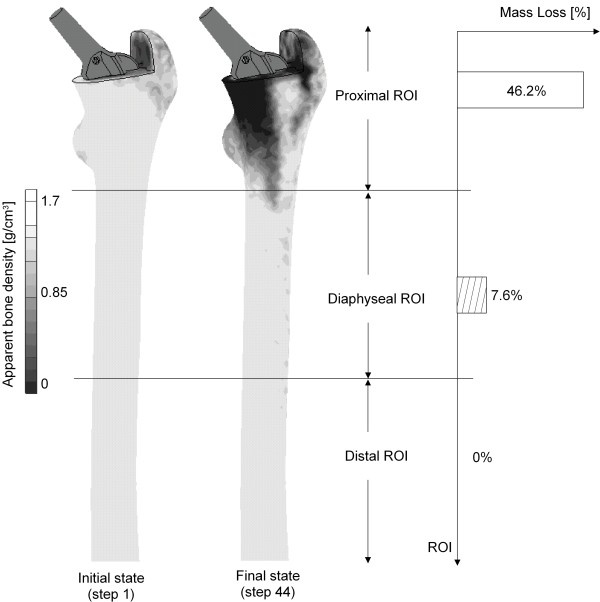
**Post-convergence distribution of the ABD in the periprosthetic femur computed with the loading regime E and calculated bone loss in the different ROIs**. Here, the ABD distribution in the frontal section of the periprosthetic femur computed with the loading regime E is shown. The initial state (step 1) in the simulation corresponds to the medical situation directly after THA, and the final state represents the results of the simulation after convergence is reached. The periprosthetic femur was subdivided into three regions of interest (ROI), a proximal, a diaphyseal and a distal one. Bone remodelling can mostly be found in the proximal ROI. The computed bone loss in this ROI is 46.2%. In the diaphyseal one, the calculated bone loss averages 7.6% and in the distal region, no bone remodelling and thus no change in the ABD occur.

Figure 8 shows the ABD distribution in the frontal section of the periprosthetic femur computed with loading regime E. The initial state (step 1) in the simulation corresponds to the medical situation directly after THA, and the final state represents the results of the simulation after reaching convergence. For a better interpretation of the results, the periprosthetic femur was subdivided into three regions of interest (ROI), a proximal, a diaphyseal and a distal one, as shown in figure 8. Herein, the proximal ROI represents the upper part of the femur and covers the BiCONTACT's coated area, the diaphyseal one covers the uncoated area of the stem, and the distal ROI describes the remaining area of the bone.

Bone remodelling can mostly be found in the proximal ROI because here the most force transmission into the bone occurs due to the coating of the stem. The computed bone loss in this ROI is 46.2%. In the diaphyseal one, although it has no severe force transmission from the prosthesis into the bone, there also occurs bone remodelling, even though it is less pronounced (calculated bone loss: 7.6%). This is probably caused by the new equilibrium position due to the remodelling in the proximal ROI. In the distal region, no bone remodelling and thus no change in the ABD occurs.

## Discussion

A current problem of THA is bone remodelling due to stress shielding, which is one of the factors causing aseptic loosening of uncemented conventional long-stem prostheses [4]. Before, this was only suspected due to biomechanical and clinical observations, but by now FEA has been established as a suitable computing method to show stress shielding by examining the decrease of the load distribution after THA. There have been several numerical investigations simulating bone remodelling via FEA because this is a time- and cost-saving as well as patient-friendly procedure which can be done in pre-clinical studies. For a realistic simulation of the bone remodelling in the periprosthetic femur, there are several important factors that have to be taken into account: the load situation, the physiological boundary conditions, the muscle forces, an appropriate bone adaptation model, realistic modelling of the composite and the mechanical properties of the bone.

Concerning the load situation, it was our aim to find out how well the loading of a complete gait cycle can be represented by a static simulation. This is why we examined several loading cases and then considered the whole gait cycle in the loading regime, and the results vary widely. They show that the examination of all loadings of a gait cycle is necessary for a realistic computation of the bone remodelling.

Other researchers [7-10,13,19,30,31] have considered two loading cases of the gait cycle and another one from stair-climbing for the simulation. According to Morlock [16], the frequency of walking is at 10.7% of the patient activity, while the frequency of stair-climbing is only at 0.7%. Therefore, walking is 15 times more frequent than stair-climbing, and accordingly bone remodelling is much more affected by the former than by the latter.

The load situation of loading case A was suggested for pre-clinical testing by Heller et al. [27]. Our results show that this loading case corresponds best to the loading regime E, with a deviation of only 14%.

Bitsakos et al. [6] did similar investigations, but only with static loading cases at 10%, 30% and 45% of the gait cycle. The influence of the load situation, however, was much greater because on the one hand there was no constraint at the femur head and on the other hand only the upper proximal part was regarded. Moreover, there was no comparison to results of a computation with the loading regime of the complete gait cycle.

Many researchers [6-10,13] also examined only the proximal part of the femur in their numerical investigations. According to Duda et al. [24] and Polgár et al. [25] this does not correspond to the physiological facts. Thus, we have modelled the whole femur in our study, as has been done in other works [11,19], in order to represent the physiological situation more realistically.

Before, none of the numerical investigations regarded the constraints according to Speirs et al [20]. We, however, did this for several reasons. Not only do they reflect the physiological constraints, but when the femur head is unconstrained the numerical simulation also yields displacements of 21 mm, which is unrealistic. A standardisation of the boundary conditions is crucial because by this the results and studies become comparable.

Furthermore, the application of the muscle forces is highly relevant for a correct computation of the load distribution in the bone and the bone remodelling after THA. In several studies it was assumed that the muscle forces may be pooled into one force with the greater trochanter as the acting point [4,7-10,13]. In our study, we examined the reduced muscle system according to Heller et al. [27]. Goetzen et al. [31] used the same muscle system in their investigations, while Taylor et al. [11] took all muscle forces into account. The latter was not done in our study for two reasons: first, to save modelling and computation time, and, second, because the other muscle forces are negligible compared to the muscle forces considered.

For the bone adaptation model, we extended the model described by Huiskes et al. [7,9,10] because it had no upper bound for the bone formation rate, which of course does not correspond to the physiology. Furthermore, we included the area of necrosis according to the findings of numerous clinical investigations, the one by Wolff [3] in particular. This modified model reflects the physiological situation much better.

Regarding the bone remodelling with loading regime E, there is more bone mass loss in the proximal ROI and much less in the diaphyseal one. This is caused by the force transmission within the prosthesis due to the proximal area being coated, which corresponds to the results of clinical studies using the same prosthesis [32].

Many investigations are done with homogeneous elastic properties for cancellous and cortical bone [11,19,30,31]. This does not correspond to reality either, for the ABD varies considerably, as shown in several clinical and experimental investigations. In our study, we thus computed the elastic modulus depending on the ABD, as other researchers have done [7-10,13,30].

In summary, the research work as described above is increasingly establishing FEA as a reliable *in silico *method for demonstrating bone remodelling due to stress shielding. However, accompanying clinical examinations are necessary to validate the computations and models used and to improve the material laws [33]. Therefore, DEXA (Dual Energy X-Ray Absorptiometry) investigations are being carried out in the Department of Orthopaedics Department of Orthopaedics of the MHH on 25 patients, provided with the uncemented long-stem BiCONTACT femur component.

## Conclusion

The objective of this study was to numerically compare the evolution of the ABD and the mass loss in the periprosthetic femur under separate static load cases of the gait cycle with their counterparts under all loading situations in this cycle. The numerical investigations prove that the FE calculation of the bone remodelling under consideration of the whole gait cycle leads to strong deviations in the mass loss and ABD distribution in comparison to the FE calculations using static loading cases. For the standardisation of the FE calculation of the bone remodelling is the consideration of all loadings in the gait cycle essential.

## Abbreviations

*D*: Strain energy density; DEXA: Dual Energy X-Ray Absorptiometry; *E*: Young's modulus of the bone; *HU*: Hounsfield Unit; *S*: Strain energy per unit of mass; *S*_*ref*_: Strain energy per unit of mass in the physiologically intact femur; *S*_*pro*_: Strain energy per unit of mass in the periprosthetic femur; STL: Standard Triangulation Language; *ρ*: Apparent bone density (ABD); : Average apparent bone density in the periprosthetic femur at the end of each computation step *n*; : Difference between average density in the prosthetically treated femur between two steps *n *- 2 and *n *- 1; : Difference between average density in the prosthetically treated femur between two steps *n *- 1 and *n*; : Apparent bone density evolution rate; : Strain vector; : Transposed stress vector; *ξ*: Stimulus for the bone remodelling; *y*: Threshold level of the necrosis in the bone adaptation law; *z*: Dead zone in the bone adaptation law.

## Competing interests

The authors declare that they have no competing interests.

## Authors' contributions

AB designed the study, carried out the numerical investigations, and prepared the manuscript. BAB, IN, PW and CSC designed the study concept from a technical and medical perspective, and corrected the manuscript.
